# Impaired mnemonic discrimination in children and adolescents at risk for schizophrenia

**DOI:** 10.1038/s41537-023-00366-9

**Published:** 2023-06-21

**Authors:** Aslıhan İmamoğlu, Christopher N. Wahlheim, Aysenil Belger, Kelly S. Giovanello

**Affiliations:** 1 Department of Psychology and Neuroscience, University of North Carolina at Chapel Hill, Chapel Hill, US; 2 Department of Psychology, University of North Carolina at Greensboro, Greensboro, US; 3 Department of Psychiatry, University of North Carolina at Chapel Hill, Chapel Hill, US; 4 Biomedical Research Imaging Center, University of North Carolina at Chapel Hill, Chapel Hill, US

**Keywords:** Human behaviour, Learning and memory

## Abstract

People with schizophrenia and their high-risk, first-degree relatives report widespread episodic memory impairments that are purportedly due, at least in part, to failures of mnemonic discrimination. Here, we examined the status of mnemonic discrimination in 36 children and adolescents (aged 11–17 years) with and without familial risk for schizophrenia by employing an object-based recognition task called the Mnemonic Similarity Task (MST). The MST assesses the ability to discriminate between studied images and unstudied images that are either perceptually similar to studied images or completely novel. We compared 16 high-risk, unaffected first-degree relatives of people with schizophrenia, bipolar disorder, and/or schizoaffective disorder to 20 low-risk, control participants. High-risk participants showed worse mnemonic discrimination than low-risk participants, with no difference in recognition memory or perceptual discrimination. Our findings demonstrate that mnemonic discrimination deficits previously observed in people with schizophrenia are also present in their young, high-risk, first-degree relatives.

## Introduction

Episodic memory, conceptualized as a memory for objects and events that are tied to a specific space and time, is fundamental to adaptive day-to-day functioning^1^. Widespread episodic memory impairments are prevalent in people with schizophrenia (see ref. ^2^ for a review) and their first-degree relatives (i.e., parents, offspring, siblings^3,4^) who exhibit higher rates of psychosis compared to the general population^5,6^ and are considered to be at familial high risk for schizophrenia. People at familial risk for schizophrenia demonstrate deficits during encoding and retrieval of episodic memories observed during free recall (e.g., refs. ^7–10^) and associative recognition (e.g., refs. ^11–13^), yet are unimpaired on tasks of item recognition^14–16^. While prior accounts have argued that these impairments reflect a differential impairment of recollection^17,18^; (for a review, see Libby et al.^19^) or context processing^14^; (for a review, see Barch & Ceaser^20^), less work has examined how mechanisms supporting the ability to encode episodic information as distinct from existing memories contribute to the impairments commonly observed in high-risk relatives.

An influential model of schizophrenia-related episodic memory deficits postulates that such impairments are, at least in part, due to failures of pattern separation^21^. Pattern separation supports the identification and subsequent organization of perceptually similar inputs into distinct, non-overlapping mnemonic representations^22^. Pattern separation occurs when a sensory input is similar but not identical to previously encountered events, thereby reducing interference of overlapping experiences^23^^,24^. Failure to engage in pattern separation can lead to false recognition that reduces one’s ability to distinguish between present and past experiences. Considering the high prevalence of false memories in schizophrenia^25^, it is paramount to determine if pattern separation is altered in high-risk relatives who may develop schizophrenia in the future.

The behavioral proxy of pattern separation, known as mnemonic discrimination, is commonly investigated by employing an object-based recognition task referred to as the Mnemonic Similarity Task (MST)^26^. The standard study-test variant of the object-based MST consists of a study phase in which participants make indoor versus outdoor judgments about images of everyday objects, followed by a recognition test phase during which participants view three types of stimuli. The test stimuli include exact repetitions of one set of studied objects (i.e., targets), objects that are perceptually similar but not identical to another set of studied objects (i.e., lures), and a set of entirely novel objects (i.e., foils). At test, participants are instructed to classify each object using one of three response options: “old” for targets, “similar” for lures, and “new” for foils. Mnemonic discrimination occurs when participants classify lures as “similar” instead of mistaking lures for targets by responding “old” (i.e., false alarms) or novel foils by responding “new” (i.e., misses). This task design also enables simultaneous examination of traditional recognition that occurs when participants correctly classify targets as “old” instead of “similar” or “new”^27^.

Prior studies employing the MST have shown mnemonic discrimination deficits in patients with first-episode psychosis^28^ and chronic schizophrenia^29,30^. These deficits presented as disproportionately poorer identification of lures as “similar.” Additionally, some studies reported traditional recognition deficits in patients^28,30^, suggesting that the mnemonic discrimination deficits in this population may reflect a general recognition deficit. To date, only one study has directly examined the relationship between mnemonic discrimination and traditional recognition in people with chronic schizophrenia^30^ using a mediation analysis. Traditional recognition deficits mediated the relationship between diagnosis status and mnemonic discrimination. Perceptual discrimination, the ability to discern between studied and lure objects while both are present in working memory, partially mediated the relationship between diagnosis and mnemonic discrimination, highlighting that other aspects of cognition play a role in schizophrenia-related differences in mnemonic discrimination.

In the current study, we examined mnemonic discrimination and its relationship to traditional recognition and perceptual discrimination in children and adolescents (11–17 years old) with or without familial risk for schizophrenia. Most people with schizophrenia spectrum disorders are diagnosed in late adolescence and early adulthood^31^. We, therefore, recruited first-degree relatives younger than the age of typical onset to assess whether changes in mnemonic discrimination and recognition are present in high-risk children. We compared high-risk participants to age- and education-matched control participants (i.e., low-risk) who had no current clinical diagnoses or familial history of psychosis.

**Fig. 1:**
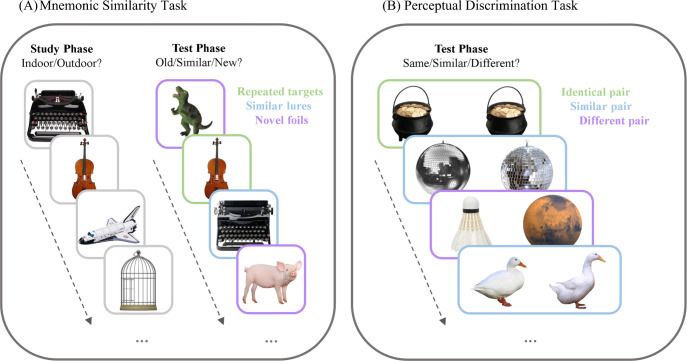
Visual depiction of the behavioral tasks. **A** The Mnemonic Similarity Task consisted of two phases: study and test. During the study phase, participants provided indoor/outdoor judgments of images of everyday objects. During the test phases, they viewed either (1) repeated targets that were identical to studied images, (2) similar lures that were perceptually similar to studied images, or (3) novel foils that had not been studied before. For each image, participants made old/similar/new judgments. **B** In the perceptual discrimination task, participants viewed pairs of images that were either identical, similar, or different from one another. For each pair, participants were asked to indicate whether the pairs were same, similar, or different from one another.

Participants completed an object-based study-test version of the MST followed by a perceptual discrimination task including object images from the MST. The MST consisted of three separate experimental study-test cycles. During the study, participants made indoor/outdoor judgments about everyday objects. At test, participants viewed exact repetitions of some studied objects (i.e., targets), perceptually similar but not identical repetitions of other studied objects (i.e., lures), and novel objects with different identities than studied objects (i.e., foils). For each object, participants made “old,” “similar,” or “new” judgments to indicate targets, lures, and foils, respectively (Fig. 1A). We subtracted false alarms from correct classifications to estimate bias-corrected mnemonic discrimination (p|Similar [Lures] - p|Similar [Foils]) and traditional recognition (p|Old [Targets] - p|Old [Foils]). Finally, participants completed a perceptual discrimination task (PDT; Fig. 1B) in which their task was to identify whether image pairs comprised objects that were identical, similar, or different by responding “same,” “similar,” or “different,” respectively. We subtracted false alarms from correct classifications to estimate a bias-corrected perceptual discrimination index for similar object pairs (p|Similar [Similar] - p|Similar [Different]).

Based on prior findings showing prominent mnemonic discrimination deficits in people with schizophrenia^28–30^, we hypothesized that high-risk participants would exhibit worse mnemonic discrimination than low-risk participants on the MST. To clarify the relationship between risk status and mnemonic discrimination, we also examined traditional recognition. Consistent with our prior work^14^, we hypothesized that young high- and low-risk participants would demonstrate comparable traditional recognition. Next, we investigated the association between risk status and perceptual discrimination of similar objects. Based on our recruitment of non-psychotic, high-risk first-degree relatives, we expected comparable perceptual discrimination between risk groups. Finally, we conducted an exploratory mediation analysis to examine the relationships among mnemonic discrimination, traditional recognition, and perceptual discrimination. We anticipated one of three possible outcomes. First, consistent with Martinelli and Shergill^30^, we could observe a full mediation, where both traditional recognition and perceptual discrimination completely mediate the relationship between mnemonic discrimination and group status. Such a finding would further support the view that schizophrenia-related mnemonic discrimination deficits reflect general recognition and perceptual discrimination deficits. A second possibility is that we observe a partial mediation, suggesting that recognition and perceptual discrimination contribute to but do not completely account for risk-related mnemonic discrimination deficits. A third and final possibility is that we find no evidence in support of mediation, suggesting that familial risk uniquely predicts worse mnemonic discrimination.

## Results

### Data analysis plan

The statistical analyses were conducted using SPSS^32^ and R^33^. Whenever applicable, we used mixed effects models from the *lme4* package in R that included subjects as random intercept effects^34^. Hypothesis tests were performed with the *Anova* function from the *car* package^35^, while post hoc comparisons used the Tukey method from the *emmeans* package^36^. The standardized effect sizes were quantified using Cohen’s *d* values derived from simple linear regression models obtained using the *eff_size* function from the *emmeans* package. The level for significance was set at α = 0.05.

As described above, we estimated mnemonic discrimination by calculating a lure discrimination index (LDI), computed as the difference between “similar” responses to lure and foil objects. We also estimated traditional recognition memory by computing the difference between “old” responses to target and foil objects. Finally, we estimated perceptual discrimination by calculating a perceptual discrimination index (PDI), computed as the difference between “similar” responses to similar and different object pairs. These indices all account for the extent that participants are biased toward providing a particular response.

To examine whether recognition and/or perceptual discrimination mediated the relationship between Risk Group (which includes people at high and low risk) and LDI, we performed a mediation analysis using the PROCESS macro Version 3.5^37^ in SPSS, that uses Preacher and Hayes’^38^ bootstrapping methodology. We based our results on 5000 bootstrap samples with bias correction and 95% confidence intervals (95% CI).

### Mnemonic Similarity Task

#### Lure discrimination

**Fig. 2:**
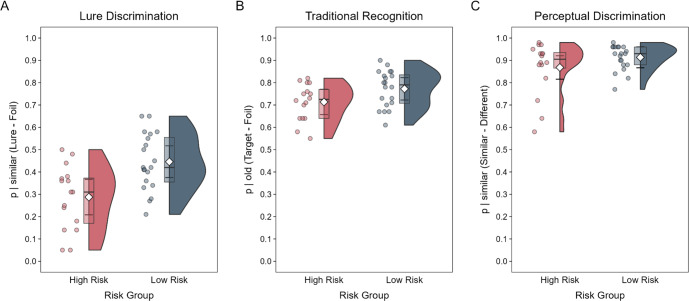
Task performance across measures. **A** The lure discrimination index was lower for the high- compared to the low-risk group. The two groups were not significantly different in **B** traditional recognition or **C** perceptual discrimination. Group means are shown as the heights of white diamonds, and error bars are 95% confidence intervals. Medians and interquartile ranges are displayed in boxplots. Distributional information is shown as individual participant estimates (dots) and the frequencies of those estimates (the width of corresponding half violin plots).

**Fig. 3:**
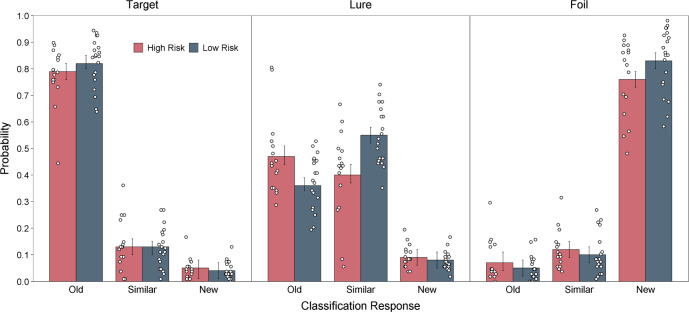
Percent endorsed for the Risk Groups for each stimulus (Target, Lure, Foil) and response type (Old, Similar, New) on the Mnemonic Similarity Task. Group means are shown as the heights of bars, and error bars are 95% confidence intervals.

We first tested our primary hypothesis that people at high familial risk for schizophrenia would show impaired mnemonic discrimination. A model including Risk Group and Experiment Run (which includes the three study-test cycles) as fixed effects indicated a significant effect of Risk Group on mnemonic discrimination, *χ*^2^(1) = 9.35*, p* < 0.01, such that the LDI score was lower for the high- than low-risk group, *d* = 0.96, 95% CI = [0.55, 1.37] (see Fig. 2A). There was no significant effect of Experiment Run, *χ*^2^(2) = 5.24*, p* = 0.07. However, there was a significant Risk Group × Experiment Run interaction, *χ*^2^(2) = 6.95*, p* = 0.03, such that the risk groups did not significantly differ in LDI on the second run of the experiment, *t*(51.6) = −1.59, *p* = 0.12. We then investigated the basis for LDI differences by conducting theoretically motivated comparisons of classifications for specific object types (see Fig. 3). High-risk participants were significantly less likely than low-risk participants to classify lures as similar, *t*(34) = −2.96, *p* < 0.01, and were more likely to identify lures as old, *t*(34) = 2.39, *p* = 0.02. There was no significant group difference in the classification of foil items as similar, *t*(33.9) = 0.83, *p* = 0.41.

We next tested the exploratory hypothesis that mnemonic discrimination deficits would vary depending on the degree of similarity between lure objects and their studied counterparts. The material set from which we selected our stimuli includes lures and studied objects varying widely in perceptual similarity to each other, indexed as the normative probabilities of participants classifying lures as studied objects^26^. Here, we selected items from the first three (out of five) lure “bins” that include lure and studied objects with the relatively greatest similarity between them. The similarity ratings range from more to less similar going from Bin 1 to Bin 3. We included objects from these bins to provide the most stringent test of mnemonic discrimination differences between risk groups, assuming that higher similarity lures place heavier demands on pattern separation. However, based on prior work showing selectivity in group-related mnemonic discrimination impairment^26^, we hypothesized that risk-related mnemonic discrimination deficits would be absent or less pronounced in Bin 1 (the most similar) than in Bins 2 and 3 (the relatively less similar) because the high similarity in Bin 1 would be challenging even for participants without risk-related memory impairment.

**Fig. 4:**
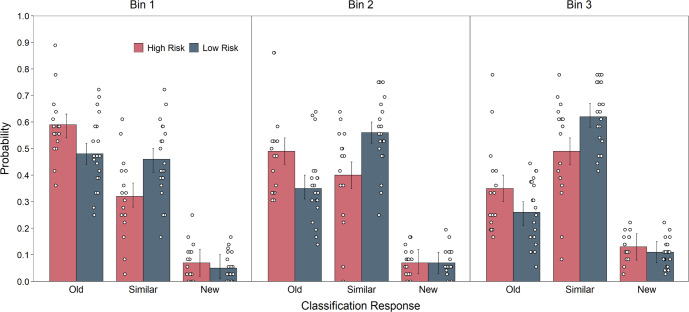
Percent endorsed for the low- and high-risk groups for each lure bin and response type on the Mnemonic Similarity Task for similar lures. Group means are shown as the heights of bars, and error bars are 95% confidence intervals.

Figure 4 displays the response probabilities for similar lures separately across each of the three lure bins. We assessed group differences among these probabilities using separate models that included Risk Group and Experiment Run as fixed effects. High-risk participants were significantly less likely than low-risk participants to classify lures as similar in all Bins, smallest *t*(33.8) = −2.46, *p* = 0.02, and more likely to classify lures as old in Bins 1 and 2, smallest *t*(33.6) = 2.23, *p* = 0.03. Furthermore, a model including Risk Group and Lure Bin as fixed effects indicated a significant effect of Lure Bin on similar classifications, *χ*^*2*^(2) = 63.28*, p* < 0.001, indicating that both groups correctly classified similar lures more often as the normative similarity between lures and studied items decreased from Bin 1 to Bin 3, smallest *t*(272) = 3.64, *p* < 0.001. A model with the same fixed effects examining old classifications indicated a significant effect of Lure Bin, *χ*^*2*^(2) = 126.43*, p* < 0.001, showing that both groups were less likely to incorrectly classify lures as old as the similarity between lures and studies items decreased, smallest *t*(272) = 5.44, *p* < 0.001. No interactions were significant, largest *χ*^*2*^(2) = 1.12*, p* = 0.57, indicating that contrary to our hypothesis, risk-related mnemonic discrimination deficits were not selective to lure bins.

#### Traditional recognition

To verify that risk-related impairment was specific to mnemonic discrimination, we then tested our secondary hypothesis that high-risk participants would show intact recognition of studied items. We compared traditional recognition memory for each risk group (see Fig. 2B) using a model including the Risk Group and Experiment Run as fixed effects. The model indicated no significant effects of Risk Group, *χ*^*2*^(1) = 2.56*, p* = 0.11, *d* = 0.58, 95% CI = [−0.12, 1.27], or Experiment Run, *χ*^*2*^(2) = 1.72*, p* = 0.42, and no significant interaction, *χ*^*2*^(2) = 1.10*, p* = 0.58, thus supporting our hypothesis. Next, we compared old response probabilities for targets and foils that contributed to the calculation of traditional recognition scores. The two groups did not differ in their old classifications of targets and foils, largest, *t*(33.9) = 1.28, *p* = 0.21.

### Perceptual discrimination task

To further verify the selectivity of the observed mnemonic discrimination deficit in people at high risk, we tested our third hypothesis that there would be no risk-related difference in bias-corrected PDI scores (Fig. 2C). A model including Risk Group and Experiment Run as fixed effects indicated no significant effects of Risk Group, *χ*^*2*^(1) = 1.72*, p* = 0.19, *d* = 0.44, 95% CI = [−0.25, 1.13], or Experiment Run, *χ*^*2*^(2) = 1.97*, p* = 0.37, on PDI, as well as no significant interaction, *χ*^*2*^(2) = 3.69*, p* = 0.16, thus supporting our hypothesis of a selective deficit in mnemonic discrimination. We then characterized the basis for the lack of risk-related PDI differences by comparing response probabilities for similar and different object pairs (see Supplementary Fig. SM1). There were no significant group differences in similar classifications to similar or different objects, largest *t*(34) = 1.44, *p* = 0.16.

### Mediation analysis

**Fig. 5:**
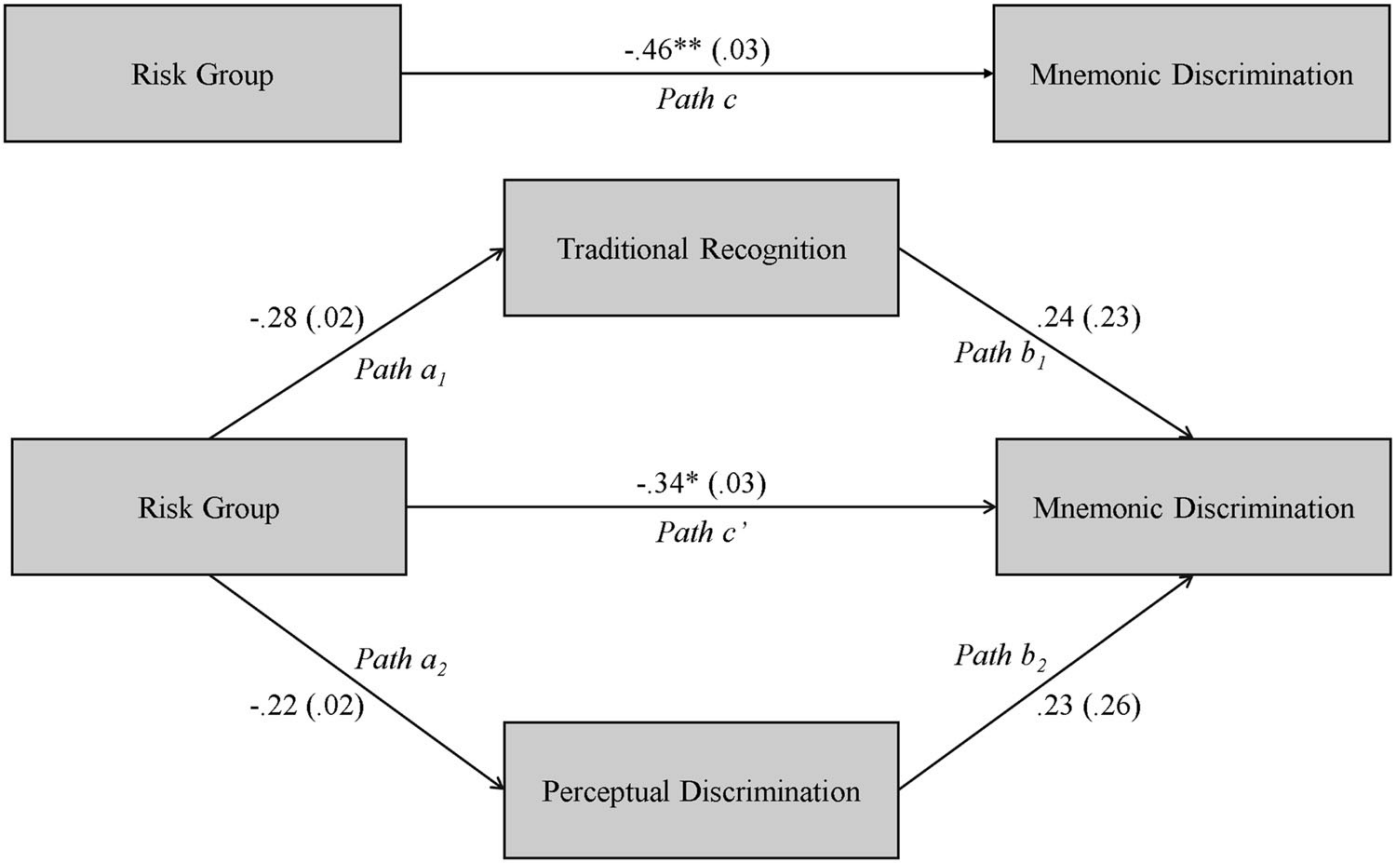
Results of the Parallel Multiple Mediator Model. The mediation analysis included risk group as the independent variable, mnemonic discrimination as the dependent variable, and both recognition memory and perceptual discrimination as parallel mediators. Partially standardized model coefficients are shown outside parentheses on relationship lines, while standard error values are in parentheses. *p < 0.05, **p < 0.01.

Previous work examining schizophrenia-related mnemonic discrimination deficits showed that both traditional recognition and perceptual discrimination mediated the relationship between clinical status and mnemonic discrimination^30^. This approach showed that diagnosis status was no longer significantly related to mnemonic discrimination after accounting for differences in traditional recognition and perceptual discrimination in separate mediation models. We conducted a similar mediation analysis to further clarify the extent to which these other aspects of cognition contributed to mnemonic discrimination. Specifically, we constructed a parallel multiple mediator model (Fig. 5), simultaneously entering traditional recognition and perceptual discrimination indices as mediators, with Risk Group as the independent variable and LDI score as the dependent variable. Given the exploratory nature of this mediation, we did not have an a priori hypothesis. However, the absence of risk-related differences in traditional recognition and perceptual discrimination suggests that those variables should not completely mediate the association between Risk Group and LDI scores.

Figure 5 shows that the Risk Group did not significantly relate to traditional recognition (Path a_1_: *p* = 0.09), and traditional recognition did not significantly relate to LDI scores (Path b_1_: *p* = 0.15). The Risk Group also did not significantly relate to PDI scores (Path a_2_: *p* = 0.20), and PDI scores did not significantly relate to the LDI scores (Path b_2_: *p* = 0.15). Finally, the Risk Group was significantly related to LDI scores (Path c: *p* < 0.01), and this relationship remained significant after accounting for traditional recognition and perceptual discrimination (Path c’: *p* = 0.03). These results suggest that mnemonic discrimination deficits observed in children and adolescents at high-risk for schizophrenia cannot be entirely explained by traditional recognition and perceptual discrimination performance.

## Discussion

The current study compared mnemonic discrimination in children and adolescents at high compared to low familial risk for schizophrenia. As hypothesized, we observed that the high-risk group was selectively impaired in mnemonic discrimination. This impairment remained when accounting for traditional recognition and perceptual discrimination abilities. These results are largely consistent with studies showing mnemonic discrimination deficits in people with first-episode psychosis^28^ and chronic schizophrenia^29,30^. We extend the existing literature by showing mnemonic discrimination deficits in young, first-degree relatives who are at heightened risk for the disorder.

High-risk participants classified similar lure items less accurately than low-risk participants, who more often made false alarms, misidentifying those items as old. This pattern of responding led to group differences in the LDI. This observation is consistent with prior studies that assessed item and response type interactions in people with schizophrenia^29,30^, first-episode psychosis^28^, and elevated negative and disorganized schizotypy^39^. However, at least one study has demonstrated lower false alarm rates of lure items in people with high positive schizotypy symptoms^40^, which can predict schizophrenia spectrum disorders^41^. Taken together, the current findings are consistent with prior research that has demonstrated a selective inability to identify lure items as similar in non-developmental populations.

To identify whether risk-related mnemonic discrimination impairment was selective to particular stimuli, we used items that varied in the degree of similarity between studied objects and lures. While past studies included up to five lure bins^26^, we specifically chose the most similar three bins that pose the most challenge for mnemonic discrimination and, thus, pattern separation. Since responding to very similar items in the first lure bin are normatively most challenging, we hypothesized that there would be no group differences for those objects. We based this hypothesis on prior findings showing that age-related mnemonic discrimination deficits occur for objects of intermediate similarity^26,42^. Inconsistent with our hypothesis, high-risk participants were less likely than low-risk participants to endorse lure items as similar, irrespective of the degree of similarity. This finding suggests that people at high familial risk for schizophrenia may be less sensitive to changes in the similarity of input. But this assertion awaits studies including lures across a wider range of perceptual similarity.

Next, we assessed the relationship between the risk group and traditional recognition memory, which captures an individual’s ability to distinguish between repeated versus novel images. As hypothesized, we demonstrated that recognition memory did not differ across risk groups. This finding is consistent with several prior studies of recognition memory in familial risk populations^14–16^. Of note, in prior studies examining mnemonic discrimination in people with schizophrenia using the MST, researchers have observed deficits in both mnemonic discrimination and traditional recognition^28,30^. In fact, one study proposed that recognition deficits seen in people with schizophrenia may underlie mnemonic discrimination^30^. This proposition was supported by a mediation analysis showing that group differences in traditional recognition and perceptual discrimination both mediated the relationship between clinical status and mnemonic discrimination^30^.

To further assess the contributions of other aspects of cognition to risk-related differences in mnemonic discrimination, we also compared high- and low-risk participants’ perceptual discrimination abilities. We employed a bias-corrected measure of perceptual discrimination, namely the PDI, to assess group differences. As hypothesized, both groups showed comparable PDI, suggesting that mnemonic discrimination differences could not be explained by perceptual abilities. Furthermore, the two groups were comparable in their likelihood of classifying similar and different item pairs as “similar.” While we did not observe a group difference in responding, there were some extreme scores in the high-risk group (see Fig. 2C), indicating that future studies with larger samples should be conducted to determine if these scores were anomalous.

Finally, we conducted a parallel mediation to test whether traditional recognition and perceptual discrimination mediated the observed relationship between risk group and mnemonic discrimination. Consistent with the absence of group differences in traditional recognition and perceptual discrimination, performance on these measures did not mediate the relationship between risk group and mnemonic discrimination. This observation is inconsistent with Martinelli and Shergill^30^, who showed that traditional recognition fully and perceptual discrimination partially mediated this relationship. The absence of a significant mediation in the current study suggests that these variables do not fully account for the schizophrenia-risk-related mnemonic discrimination deficits observed here.

While we did not observe a significant relationship between mnemonic discrimination and perceptual discrimination in our mediation analysis, we acknowledge that our study may have been underpowered to detect such an effect. Past cognitive aging literature has demonstrated that individuals’ perceptual discrimination abilities significantly relate to their mnemonic discrimination performance^43,44^. Nevertheless, we detected a significant relationship between risk status and mnemonic discrimination above and beyond these potential contributions. Given the small sample size, the present findings are best considered as preliminary evidence. Larger samples will be required in future work to verify the selective risk-related deficits reported here.

More generally, theoretical models of hippocampal function postulate that the ability to pattern separate, which may lead to mnemonic discrimination, relies critically on the integrity of the dentate gyrus (DG) and Cornu Ammonis 3 (CA3) subregions of the hippocampal formation^23,45^. Such postulations have been supported by neuroimaging studies demonstrating that CA3/DG volume significantly predicts mnemonic discrimination performance on the MST^46^. Other studies observed CA3/DG activity consistent with pattern separation when participants encountered lure items^22,47^. Notably, these subregions are structurally altered in people with schizophrenia (see ref. ^48^ for a review). While the aforementioned studies signal to the mnemonic discrimination deficits observed in schizophrenia being tied to hippocampal structure and function, our current study did not employ any neuroimaging methods that would allow us to test this hypothesis. Therefore, future studies should investigate the effect of familial risk on the neural underpinnings of mnemonic discrimination.

In conclusion, we found that mnemonic discrimination deficits commonly observed in people with schizophrenia are also present in their younger, high-risk, first-degree relatives. We observed these deficits in late childhood and early adolescence. Mnemonic discrimination is a critical component of episodic memory that is paramount for distinguishing between current perceptions and similar memory representations. The current findings thus highlight the importance of studying mnemonic discrimination in not only people with schizophrenia but also with their high-risk relatives who are at risk of developing the disorder in the future. Although beyond the scope of the current study, characterizing mnemonic discrimination deficits in at-risk youth may provide an effective instrument in predicting future propensity to generate false memories that create a susceptibility to psychosis^49^, which is a hallmark symptom of schizophrenia^50^.

## Methods

All procedures were approved by the Institutional Review Board of the University of North Carolina at Chapel Hill.

### Participants

Participants were 36 children and adolescents (16 female) 11–17 years of age. A portion of the participants were recruited from a larger study at the University of North Carolina at Chapel Hill (UNC) entitled, ‘Cognition and Neuroimaging in Teens’ (CogNIT). High-risk participants in the CogNIT pool were recruited from the Outreach and Support Intervention Services, the Schizophrenia Treatment and Evaluation Program, public schools, and community clinics, while low-risk (i.e., control) participants were recruited from the community and nearby schools through flyers and listservs. The high-risk group included 16 children and adolescents with a parent or a sibling (i.e., a first-degree relative) with a psychotic disorder diagnosis (i.e., schizophrenia, schizoaffective disorder, bipolar disorder) as these disorders share a common, underlying genetic vulnerability to schizophrenia^51,52^. The low-risk group included 20 participants with no family history of psychotic mental illnesses and no current clinical diagnoses. Diagnoses were assessed based on a modified version of the Structured Clinical Interview for DSM-IV Axis I disorders (SCID^53^). The exclusionary criteria for both groups included having any DSM-IV psychotic or mood disorders, substance abuse disorder, and/or taking any medications that directly alter cardiovascular function. Four high-risk participants with a comorbid diagnosis of ADHD were being treated with stimulant medication.

All participants provided informed consent (parent) and informed assent (children) prior to participation. Participants were compensated for their time and travel. Participants were age-matched across groups, and the mean age did not significantly differ for the groups, *t*(34) = 0.03, *p* = 0.98. Self-reported race/ethnicity represented a sample of 25 Caucasian (69.4%), eight African American (22.2%), two Hispanic (5.6%), and one Multiracial (2.8%). While each participant completed all parts of the experiment, partial MST data were included for three participants due to a computer error that resulted in missing data. These three participants were still included in the analyses since they had at least one MST experiment run, including 108 test trials, which constituted a complete set. One participant was excluded from all analyses due to having recognition scores that were 3.8 SD below the mean.

### Procedure

All data were collected on a lab computer using E-Prime software (Version 3, Psychology Software Tools)^54^. Experimental tasks were administered in a fixed order, with the MST being administered before the PDT. Details of each task are described below.

#### Mnemonic Similarity Task

The MST consisted of three distinct experimental sets, with each set containing two phases. In the first phase (i.e., incidental encoding), participants made ‘indoor’ vs. ‘outdoor’ judgments about 72 colored pictures of everyday objects that appeared one at a time (see Fig. 1A). Each image appeared for 2 s with a 0.5 s interstimulus interval (ISI). In the second phase (i.e., test), participants were given a recognition memory test in which they were shown (1) exact repetitions of images presented in the study phase (i.e., targets), (2) new images that had not been shown before (i.e., foils), and (3) images that are perceptually similar, but not identical, to those seen during the study phase (i.e., lures). For each image presented, participants made “old,” “similar,” or “new” judgments via a button press. Participants had 2 s to make these judgments with a 0.5 s ISI. Each of the three test phases consisted of 108 object images containing an equal number of targets, lures, and foils (36 per condition). Thus, each participant responded to a total number of 324 critical trials, including 108 targets, 108 lures, and 108 foils. The order in which these images appeared was counterbalanced across participants. The task also included three bins of lure items that varied in the degree of similarity from the most similar Bin 1 to the relatively less similar Bins 2 and 3. The rank ordering of these lures was based on the rates of false alarm “old” responses to lures, which was obtained from a large, independent population of young adults^26^. There were a total number of 12 objects per lure similarity bin within a given experimental set, which resulted in 36 objects per lure bin per participant. Each experimental set contained a different set of stimuli, and the order of experimental sets was counterbalanced across participants. Failures to respond within 2 s resulted in 2.35% missing observations. The percentage of missing observations was significantly different across risk groups, with the high-risk group exhibiting more missing observations (3.65%) than the low-risk group (1.32%), *t*(34) = 2.64, *p* = 0.01.

#### Perceptual discrimination task

Participants completed three versions of the PDT, which were administered in a counterbalanced order. Participants viewed 108 object images taken from the MST testing phase. Participants viewed these images in 90 pairs, which included 36 pairs of identical images, 36 pairs of similar-looking images, and 18 pairs of different images (see Fig. 1B). Participants were asked to classify the relationship within pairs by providing “same,” “similar,” or “different” responses. Responses were self-paced without a response deadline.

## Supplementary information

Supplemental Figure

## Data Availability

The data analyzed during the current study are available from the corresponding author upon reasonable request. Analysis scripts are available on the OSF: 10.17605/OSF.IO/CU95R.
