# Supplementary material for: Impaired mnemonic discrimination in children and adolescents at risk for schizophrenia
Source: Schizophrenia (Heidelb). 2023 Jun 21;9(1):39. doi: 10.1038/s41537-023-00366-9 (PMC10284829; doi:10.1038/s41537-023-00366-9)
Supplement: Supplementary file 1 — Supplemental Figure [file 41537_2023_366_MOESM1_ESM.docx]

**Supplementary Material**

**Figure SM1**

*Percent endorsed for the Risk Groups for each stimulus (Identical, Similar, Different) and response type (Same, Similar, Different) on the Perceptual Discrimination Task.*


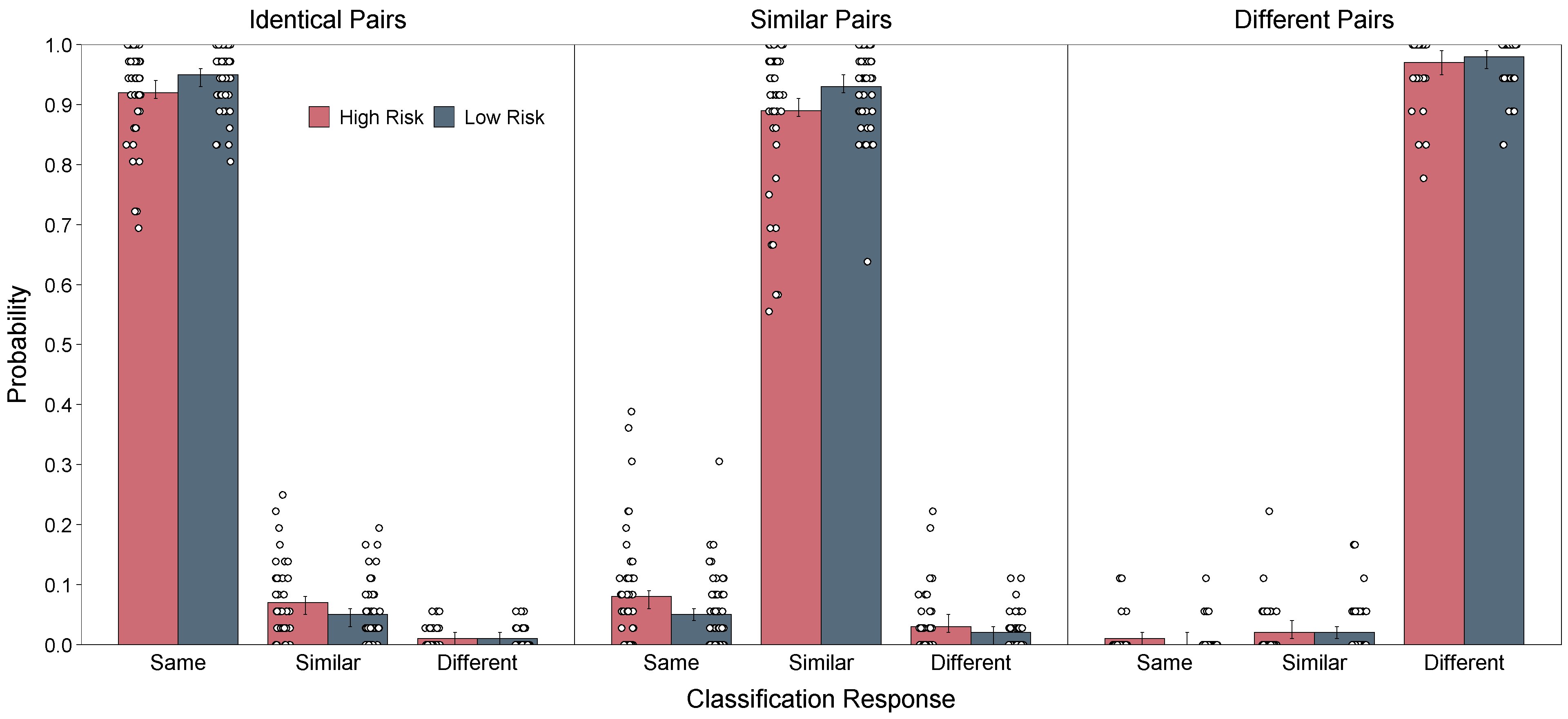


*Note.* Group means are shown as the heights of bars and error bars are 95% confidence intervals.
